# E-proteins orchestrate the progression of neural stem cell differentiation in the postnatal forebrain

**DOI:** 10.1186/1749-8104-9-23

**Published:** 2014-10-29

**Authors:** Bruno Fischer, Kasum Azim, Anahí Hurtado-Chong, Sandra Ramelli, María Fernández, Olivier Raineteau

**Affiliations:** 1 Brain Research Institute, ETH Zurich/University of Zurich, 8057 Zurich, Switzerland; 2 Stem Cell and Brain Research Institute, Inserm U846 Lyon, 69675 Bron, France; 3 Université de Lyon, Université Lyon I, 69003 Lyon, France

**Keywords:** Class I basic helix-loop-helix proteins, E-proteins, Neural differentiation, Postnatal neurogenesis, Stem cell plasticity

## Abstract

**Background:**

Neural stem cell (NSC) differentiation is a complex multistep process that persists in specific regions of the postnatal forebrain and requires tight regulation throughout life. The transcriptional control of NSC proliferation and specification involves Class II (proneural) and Class V (Id1-4) basic helix-loop-helix (bHLH) proteins. In this study, we analyzed the pattern of expression of their dimerization partners, Class I bHLH proteins (E-proteins), and explored their putative role in orchestrating postnatal subventricular zone (SVZ) neurogenesis.

**Results:**

Overexpression of a dominant-negative form of the E-protein *E47* (*dnE47*) confirmed a crucial role for bHLH transcriptional networks in postnatal neurogenesis by dramatically blocking SVZ NSC differentiation. *In situ* hybridization was used in combination with RT-qPCR to measure and compare the level of expression of E-protein transcripts (*E2-2*, *E2A*, and *HEB*) in the neonatal and adult SVZ as well as in magnetic affinity cell sorted progenitor cells and neuroblasts. Our results evidence that E-protein transcripts, in particular *E2-2* and *E2A*, are enriched in the postnatal SVZ with expression levels increasing as cells engage towards neuronal differentiation. To investigate the role of E-proteins in orchestrating lineage progression, both *in vitro* and *in vivo* gain-of-function and loss-of-function experiments were performed for individual E-proteins. Overexpression of *E2-2* and *E2A* promoted SVZ neurogenesis by enhancing not only radial glial cell differentiation but also cell cycle exit of their progeny. Conversely, knock-down by shRNA electroporation resulted in opposite effects. Manipulation of E-proteins and/or Ascl1 in SVZ NSC cultures indicated that those effects were Ascl1 dependent, although they could not solely be attributed to an Ascl1-induced switch from promoting cell proliferation to triggering cell cycle arrest and differentiation.

**Conclusions:**

In contrast to former concepts, suggesting ubiquitous expression and subsidiary function for E-proteins to foster postnatal neurogenesis, this work unveils E-proteins as being active players in the orchestration of postnatal SVZ neurogenesis.

## Background

The self-renewal versus differentiation of neural stem cells (NSCs) into new neurons is a highly complex process and depends on tight regulatory mechanisms throughout postnatal life. Early after birth, radial glia cells (RGCs) mature into type-B cells that reside in the germinal niche lining the lateral ventricles (LVs), namely the subventricular zone (SVZ). These NSCs proliferate slowly (infrequently) and by asymmetric division give rise to fast proliferating cells, defined as type-C cells (or transit amplifying progenitors). After 3 to 4 cycles of symmetric divisions, allowing the pool of type-C cells to enlarge, these progenitors start to differentiate and commit to specific fates [1]. At this stage, they are defined as type-A cells (or neuroblasts), which then migrate through the rostral migratory stream (RMS) into the olfactory bulb [2,3] to terminally differentiate into specific neuron subtypes (i.e., periglomerular neurons and granule cells) [4].

To balance NSC maintenance and precursor differentiation into neurons or glial cells, postnatal neurogenesis relies on the timely integration of intracellular signaling mechanisms such as the basic-helix-loop-helix (bHLH) transcription factor machinery. bHLH proteins represent a large family of transcription factors that are categorized in several classes based on their structure and function [5] and are believed to play an essential role in multiple aspects of forebrain development. By virtue of their transcriptional activity, bHLH proteins can be subdivided into three groups, i.e., activators (Class II), repressors (Class V), and transactivators (Class I) [6].

The classical view of bHLH protein interaction and function is still largely based on drosophila studies [7-9] and murine developmental studies [5,10-12]. There, bHLH repressors (i.e., Hes and Id proteins) and activators (proneural proteins, i.e., Ascl1, Neurog2) mutually antagonize each other either by direct transcriptional repression [13,14], physical inhibition [8,15], or by competing for bHLH transactivators (E-proteins) [12,16]. This antagonistic balance defines whether NSCs or their progeny are differentiating or not, with bHLH repressors predominantly favoring the retention of cells in immature stages [17,18], while bHLH activators accelerate progenitor maturation [13,14,19-22].

Recent work done in drosophila, however, indicates that this established paradigm is likely to be over-simplified and incomplete. A more sophisticated spatial and temporal regulation of E-protein expression has been proposed, suggesting that E-proteins (*E2-2*, *E2A*, *HEB*) fulfill a more complex function than anticipated in germinal zones of the central nervous system (CNS) [23,24]. Furthermore, work in rodents implies that various E-proteins exhibit differential patterns of expression at distinct ages, resulting in the preferential formation of specific dimers [25-27].

This complex pattern of expression, paired with a recent study implicating Ascl1 in regulating both NSC proliferation and differentiation [22], suggests that E-proteins orchestrate NSC lineage progression, a hypothesis we examine in the present study.

## Results

### Heterodimerization of Class I/II bHLH proteins is essential for normal NSC differentiation *in vitro* and *in vivo*

NS5 cell cultures contain homogenous populations of progenitors that divide indefinitely upon exposure to FGF2 and EGF and undergo neuronal and glial differentiation following mitogen withdrawal [28]. Overexpression of proneural proteins, such as Ascl1, in progenitors of various origins results in their rapid neuronal differentiation [19-22].

**Figure 1:**
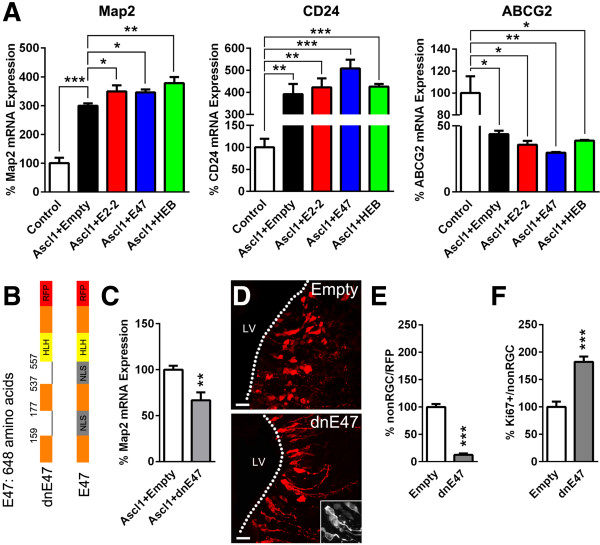
***In vitro *and *in vivo *modulation of bHLH function regulates neurogenesis. (A)***Ascl1* nucleofection in NS5 cells caused an increased *Map2* and *CD24* expression whilst conversely decreasing *ABCG2* mRNA expression, as detected via RT-qPCR (100 ± 19.1 vs*.* 299.4 ± 8.4, 100 ± 19.6 vs*.* 392.1 ± 46.1, 100 ± 15.2 vs*.* 43.8 ± 2.5, respectively). Additionally, all E-proteins (*E2-2*, *E47*, *HEB*) further potentiated *Ascl1*-induced alteration in marker expression, when co-nucleofected with *Ascl1* (*Map2*: 299.4 ± 8.4 vs*.* 349 ± 21.2, 345.7 ± 10, 378.3 ± 21; *CD24*: 392.1 ± 46.1 vs*.* 423.3 ± 39.7, 508.5 ± 40.2, 426.4 ± 11.7; *ABCG2*: 43.8 ± 2.5 vs*.* 35.7 ± 2.9, 29.7 ± 0.5, 38.8 ± 0.6, respectively). **(B)** Schematic illustration of the dominant-negative construct of *E47* (*dnE47*), where the nuclear localization sequence is missing, therefore preventing its nuclear translocation and transcriptional activity of its dimerizing partners (**F**, insert). **(C)** Co-nucleofection of *dnE47* reduced *Ascl1*-induced neurogenesis as revealed by *Map2* RT-qPCR measurements (100 ± 4.2 vs*.* 66.8 ± 8.5). **(D, E)** Targeted *in vivo* electroporation of the *dnE47-RFP* construct rapidly reduced RGC differentiation, as revealed by the lower proportion of non-RGCs, when compared to an empty RFP control plasmid (100 ± 5.5 vs*.* 12.6 ± 2.7) 2 days post-electroporation. **(F)** Cycling progenitors (non-RGC) were maintained proliferating (Ki67^+^) following *dnE47* induction (100 ± 9.6 vs. 182 ± 9.6). *P* values: *P <0.05; **P <0.01; ***P <0.001. All quantifications were normalized to control conditions. Scale bars: **D**, 20 μm.

In the first instance, the role of Class I/II bHLH factors in mediating neurogenesis was assessed *in vitro*. The efficiency of neuronal differentiation following overexpression of *Ascl1* alone or in combination with E-proteins when NS5 cells were grown in proliferative culture conditions was determined. Overexpression of *Ascl1* induced a >3-fold increase in neuronal differentiation compared to an empty control plasmid, as revealed by elevated *Map2* or *CD24* transcript expression, both of which are immature neuron markers (Figure 1A). Cotransfection of *Ascl1* with either E-protein, i.e., *E2-2*, *E47* (*E2A* isoform), and *HEB*, further potentiated *Ascl1*-induced neuronal differentiation by approximately 50 to 80% when *Map2* expression was measured, and to a lesser extent when *CD24* transcription was probed (Figure 1A). In contrast, measurement of *ABCG2*, a transcript expressed in NSCs, showed opposite trends (Figure 1A). Together, these results illustrate the interchangeability of E-proteins in this cellular context and their potential to promote *Ascl1*-induced NS5 cell differentiation.

We next disrupted Class I/II bHLH transcriptional activity *in vitro* and *in vivo* to investigate its effect on NSC differentiation. We used a mutated form of the *E2A* isoform *E47*, which lacks its nuclear localization sequence and fails to translocate to the cell nucleus, and therefore sequesters its binding partners within the cytoplasm [29,30] (Figure 1B,D (insert)). While overexpression of dominant-negative E47 (*dnE47*) alone did not influence *Map2* transcript expression in proliferative culture conditions (Additional file 1A), it efficiently prevented *Ascl1*-induced neuronal differentiation of NS5 cells as shown by the partial blockade of *Map2* induction (Figure 1C). We next tested the effect of *dnE47* in SVZ NSCs (i.e., radial glia cells (RGCs) at this early postnatal stage) by performing postnatal *in vivo* electroporation. Early after birth, NSCs can be easily distinguished from their progeny based on morphological criteria, i.e., an elongated cell body and the presence of a basal and apical process [31,32]. Quantification revealed a dramatic blockade of differentiation following *dnE47* overexpression, with most electroporated RFP^+^ cells still presenting a clear RGC morphology (Figure 1D,E). Interestingly, cells that were already undergoing differentiation into non-radial glial cells (non-RGCs) exhibited an enhanced proliferative phenotype, as demonstrated by the doubling of the number of Ki67^+^/RFP^+^ non-RGCs (Figure 1F, Additional file 1B).

**Figure 2:**
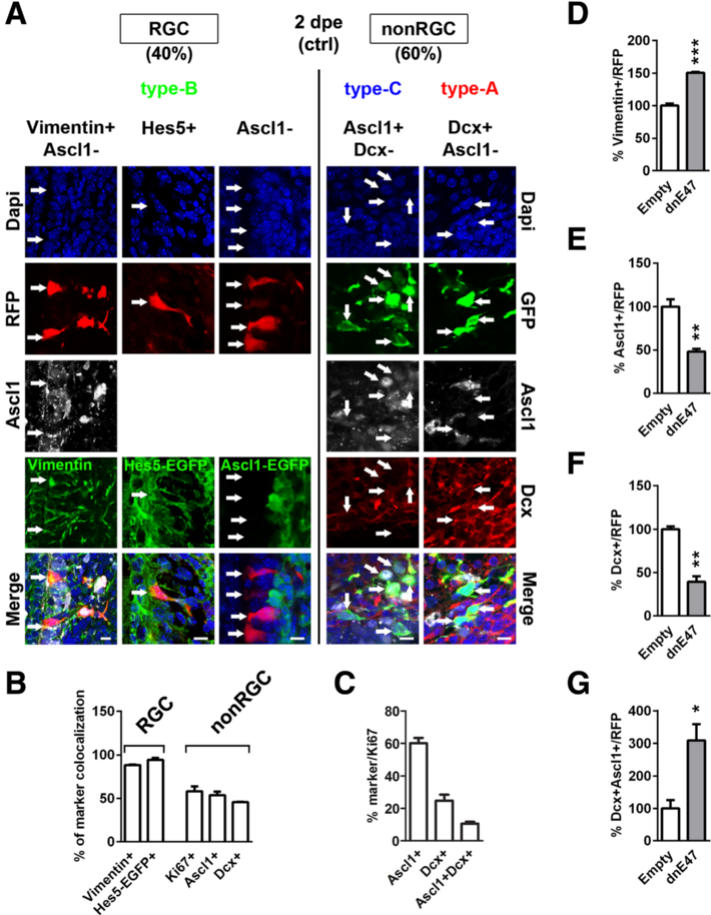
**Antigenic properties of radial glial as well as non-radial glial cells and confirmation of differentiation blockade by *dnE47 *overexpression. (A, B)**, At 2 days post electroporation (dpe), 40% of the labeled cells showed morphological criteria of radial glial cells (RGCs, i.e., bipolar processes, apical connection to lateral ventricle (LV) lumen), while 60% presenting a spherical morphology were distant from the LV and were defined as non-RGCs. Morphologically-defined RGCs express the NSC marker Vimentin (88.4 ± 0.7) and were EGFP positive in Hes5-EGFP reporter mice (94.2 ± 2.4), but negative in Ascl1-EGFP reporter mice, in which type-B or type-C cells are labelled, respectively [33,34]. In contrast, non-RGCs consisted of a heterogeneous population of type-C (i.e., Ascl1^+^, 53.6 ± 4.3) and type-A cells (i.e., Dcx^+^, 45.9 ± 0.2). Non-RGCs were frequently proliferative (i.e., Ki67^+^, 58.2 ± 5.7). **(C)** A subsequent characterization of cycling non-RGCs revealed that most Ki67^+^ cells were type-C cells (Ascl1^+^, 60.1 ± 4.4), whereas fewer were type-A cells (Dcx^+^, 22.9 ± 4.0). Notably, a minor population of cycling cells were double-positive for Ascl1 and Dcx (10.1 ± 1.4), probably representing a transition phase in between type-C and type-A phenotypes. **(D–G)** Antigenic assessment of RGC differentiation blockade following *dnE47* overexpression. Electroporation of *dnE47* in the postnatal SVZ reduced RGC differentiation, as revealed by the increased number of Vimentin^+^/RFP^+^ RGCs **(D)** and concomitant decrease of Ascl1^+^/RFP^+^**(E)** and Dcx^+^/RFP^+^**(F)** non-RGCs, respectively, when compared to an empty RFP control plasmid 2 dpe (Vimentin: 100 ± 3.2 vs*.* 150.5 ± 1.6; Ascl1: 100 ± 8.4 vs*.* 48.2 ± 3.1; Dcx: 100 ± 3.3 vs*.* 39.5 ± 6.3). This blockade of differentiation was further supported by an increase in Dcx^+^/RFP^+^ type-A cells retaining expression of the type-C cell marker Ascl1 (Dcx^+^Ascl1^+^, 100 ± 25.7 vs*.* 308.5 ± 50.3) **( *G *)**. *P* values: *P <0.05; **P <0.01; ***P <0.001. Quantifications were normalized to control conditions **(D–G)**. Scale bars: **A**, 10 μm.

To confirm the accuracy in monitoring RGC differentiation progression by electroporation and analysis of morphological criteria, we next performed an in depth antigenic characterization of RGCs and non-RGCs. At 2 days post-electroporation (2 dpe), RGCs were highly positive for type-B cell markers (i.e., Vimentin and Hes5-EGFP) and completely devoid of the type-C cell marker Ascl1 (Figure 2A,B). In contrast, non-RGCs were characterized as a mix of type-C (Ascl1^+^, 50%) and type-A (Dcx^+^, 50%) progenitors (Figure 2A,B). Approximately half of the non-RGCs were proliferating, as indicated by expression of Ki67 (Figure 2B). Those proliferating cells were mostly Ascl1^+^ type-C cells (~60%, Figure 2C, Additional file 1C), while only ~25% expressed the type-A cell marker Dcx (Figure 2C, Additional file 1D). Interestingly, ~10% of proliferating non-RGCs exhibited a transitory phenotype between type-C and type-A cell stages and were positive for both markers (Ascl1^+^Dcx^+^; Figure 2C).

We next confirmed the blockade of RGC differentiation after ectopic *dnE47* using these antigenic criteria. Overexpression of *dnE47* resulted in an increase in the number of Vimentin^+^/RFP^+^ cells (Figure 2D, Additional file 1E), whilst the numbers of Ascl1^+^/RFP^+^ (type-C) and Dcx^+^/RFP^+^ (type-A) precursors were concomitantly decreased at 2 dpe compared to controls (Figure 2E,F, Additional file 1F,G). Strikingly, the retention of a more immature phenotype by the electroporated cells was further supported by the 3-fold increase of Dcx^+^ cells, still expressing type-C cell specific Ascl1 (Figure 2G).

Altogether, these experiments demonstrate that the progression of RGC differentiation can be analyzed based on morphological criteria and reveal a dependence of NSC differentiation on Class I/II bHLH transcriptional activity in the postnatal SVZ.

### E-proteins exhibit a complex spatio-temporal pattern of expression in the postnatal forebrain

**Figure 3:**
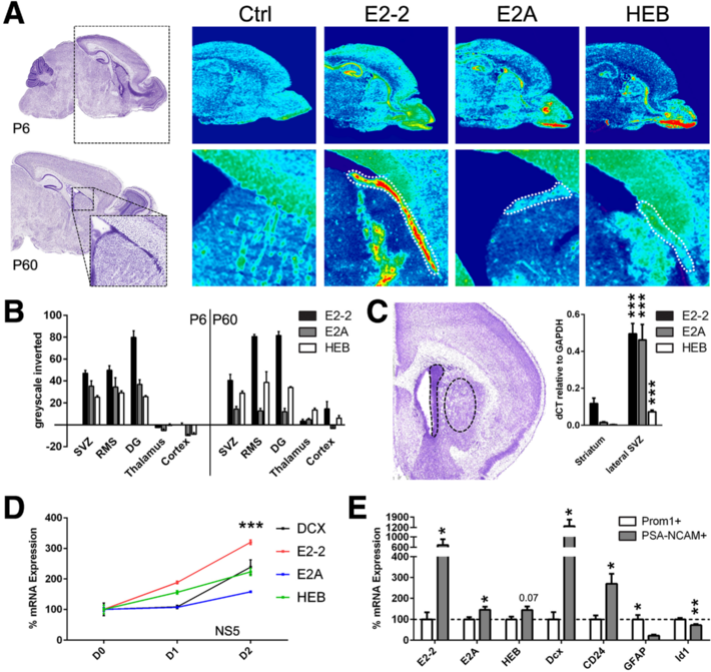
**E-protein mRNA expression is enriched in germinal regions of the postnatal and adult forebrain and is increased during NSC differentiation *in vitro *and *in vivo*. (A)** ISH of *E2-2*, *E2A*, and *HEB* revealed its enhanced expression in the SVZ (dashed zone at P60), the RMS, and the dentate gyrus, when compared to non-neurogenic regions (i.e., thalamus, cortex) at P6 as well as P60. **(B)** Inverted greyscale quantification of **A***.***(C)***E2-2*, *E2A*, and *HEB* transcripts were highly expressed in the lateral SVZ compared to the striatum (dashed zone circling both regions) (0.18 ± 0.03 vs*.* 0.5 ± 0.06, 0.02 ± 0.004 vs*.* 0.46 ± 0.08, 0.003 ± 0.0004 vs. 0.07 ± 0.01, respectively). **(D)** Upon differentiation, E-protein mRNA levels were upregulated in NS5 cells (two-way ANOVA followed by Dunnett’s *post hoc* test). Differentiation progress was confirmed by upregulation of *doublecortin* (*Dcx*) transcripts, an early neuronal marker, and normalized to *GAPDH* and shown as a percentage vs. D0. **(E)** E-protein transcription, in particular *E2-2*, was enhanced in more mature PSA-NCAM^+^ MAC sorted neuroblasts compared to earlier Prominin-1^+^ (or CD133^+^) NSCs/progenitors (100 ± 32.0 vs*.* 687.8 ± 226.4, 100 ± 9.9 vs*.* 145.4 ± 15.4, 100 ± 12.1 vs*.* 144.0 ± 17.4, respectively). Cell sorting efficiency was confirmed by enrichment of *Dcx* (100 ± 34.0 vs*.* 1291.3 ± 438.6) and *CD24* (100 ± 17.8 vs*.* 270.1 ± 48.1) transcripts in neuroblasts, while the generic NSC transcripts *GFAP* (100 ± 20.4 vs*.* 22.1 ± 4.5) and *Id1* (100 ± 6.5 vs*.* 72.8 ± 4.7) were enriched within Prominin-1^+^ sorted cells. *P* values: *P <0.05; **P <0.01; ***P <0.001. All quantifications were normalized to control conditions **(D, E)**.

Based on these findings, we further explored both spatial and temporal E-protein expression in the postnatal and adult forebrain. We first analyzed the spatial pattern of expression by *in situ* hybridization (ISH) at postnatal day 6 (P6) and 60 (P60). All three genes coding for E-proteins (*E2-2*, *E2A*, and *HEB*) were abundant in the postnatal forebrain and were enriched in germinal zones, i.e., dentate gyrus, SVZ, and RMS at P6 (Figure 3A,B). These results were confirmed by real-time quantitative polymerase chain reaction (RT-qPCR) measurement from the microdissected lateral SVZ and adjacent parenchyma (i.e., striatum). All E-protein transcripts were enriched in the SVZ, with a clear predominance of *E2-2* and *E2A* (Figure 3C). At P60, E-protein transcripts were generally less abundant compared to earlier postnatal ages. While *E2-2* expression was still enhanced in the SVZ and the RMS, *E2A* and *HEB* expression levels were only marginally higher than in non-germinal regions (Figure 3A,B), thus implying that *E2-2* may be required for neuronal differentiation continually during development and into adulthood.

Having demonstrated an enrichment of E-protein transcripts in germinal regions of the postnatal forebrain, regulation during early neuronal differentiation was assessed. *In vitro* differentiation of the NS5 cell line increased the expression of all E-protein transcripts. Upregulation of *E2-2* and *HEB* had occurred following 24 h, before any increase of *doublecortin* (*Dcx*), a neuronal differentiation marker, could be detected. After 48 h of differentiation, all E-protein transcripts were significantly upregulated (Figure 3D).

A similar upregulation was observed *in vivo*. Early progenitors and committed neuroblasts were isolated based on their differential expression of the cell surface markers Prominin-1 and PSA-NCAM, respectively. Measurements of cell-specific markers confirmed the efficiency of the approach by showing that Prominin-1^+^ sorted cells were enriched for the stem cell markers *GFAP* and *Id1*, while *Dcx* and *CD24* enrichment was prominent in PSA-NCAM^+^ sorted cells. Measurements of E-protein transcripts by RT-qPCR revealed that *E2-2* and, to a lesser extent, *E2A* and *HEB*, were strongly upregulated in the postnatal SVZ during NSCs differentiation (Figure 3E).

Altogether, our results indicate that E-protein expression is spatially and temporally regulated in a pattern that matches NSC differentiation during postnatal forebrain development.

### E-protein manipulation alters the progression of NSC differentiation in the postnatal SVZ

**Figure 4:**
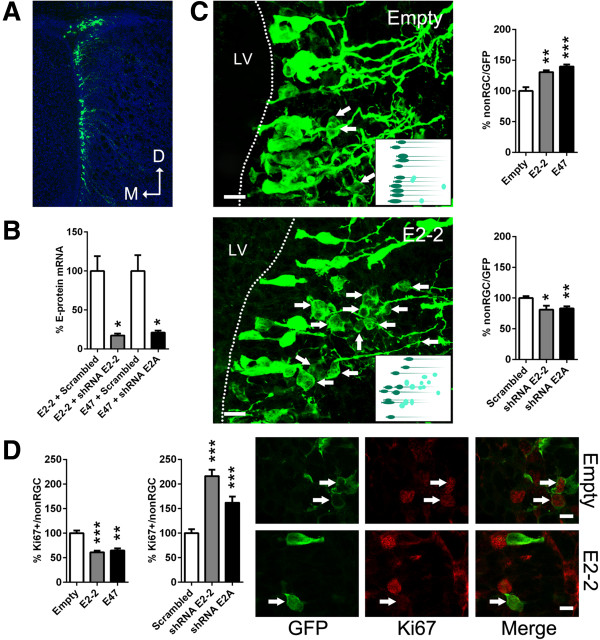
***E2-2 *and *E2A *sequentially affect RGC differentiation and progenitor proliferation. (A)** Representative illustration of a green fluorescence protein (GFP) plasmid electroporation into the lateral wall of the LV. **(B)***In vitro* characterization of the shRNAs used in this study. shRNA against *E2-2* or *E2A* decreased *E2-2* or *E2A* expression (100 ± 18.9 vs. 17.2 ± 2.4 or 100 ± 20.2 vs. 20.9 ± 2.5, respectively) within neurosphere cultures, when co-nucleofected with *E2-2* or *E47* overexpression plasmids, respectively. **(C)***E2-2* and *E47* overexpression constructs increased transition from RGCs (bipolar processes, apical connection to LV lumen) to non-RGCs (no processes, spherical morphologies, distant from the LV wall) at 2 dpe (100 ± 3.9 vs. 130.5 ± 3.0 or 139.7 ± 1.4, respectively). When shRNA plasmids against *E2-2* or *E2A* were applied, most electroporated cells maintained a RGC morphology, as reflected by the reduced ratio of non-RGC cells compared to control conditions (100 ± 3 vs. 81 ± 6.4 or 82.9 ± 3.6, respectively). Confocal micrographs illustrate RGCs and non-RGCs coexisting in the neurogenic niche. Inserts show a schematic view of the RGC to non-RGC transition. **(D)***E2-2* and *E47* overexpression constructs decreased Ki67 immunoreactivity within progenitors (non-RGC) at 2 dpe (100 ± 5.6 vs*.* 61 ± 3.5 or 64.7 ± 4.1, respectively). shRNA constructs against *E2-2* and *E2A*-induced opposite effects (100 ± 8 vs*.* 216 ± 13.2 or 161.9 ± 12.7, respectively). Confocal micrographs illustrate representative non-RGCs positive or negative for Ki67, respectively. *P* values: *P <0.05; **P <0.01; ***P <0.001. All quantifications were normalized to control conditions. Scale bars: **C**, 20 μm; **D**, 10 μm.

We next performed further E-protein gain-of-function (GoF) and loss-of-function (LoF) experiments in the postnatal lateral SVZ, by means of plasmid electroporation (as above) (Figure 4A). We focused this functional characterization onto *E2-2*, which appears to be the most prominent E-protein expressed in the postnatal and adult SVZ (see above), and *E2A*, which is the best studied E-protein in CNS development [23,25,29,35-37].

GoF experiments resulted in the rapid transformation of RGCs into non-RGCs. Thus, whilst *E2-2* and *E2A* overexpression increased the transformation of RGCs into non-RGCs by 30% and 40%, respectively, both *E2-2* and *E2A* silencing decreased it by 20% (Figure 4B,C, Additional file 1H). We next focused our analysis on non-RGCs, which, at this early timepoint (2 dpe), consisted of transient amplifying progenitors (type-C) and neuroblasts (type-A) (Figure 2A,B). E-protein overexpression significantly reduced the proliferation of non-RGCs, as demonstrated by a ~45% decrease in the number of green fluorescence protein positive (GFP^+^) non-RGCs expressing the cell cycle marker Ki67. In contrast, LoF enhanced the proliferation capacity of non-RGCs (Figure 4D). Interestingly, these effects were more pronounced following *E2-2* silencing, consistent with its higher expression in the postnatal SVZ compared to *E2A* (see above).

Altogether, these data are in agreement with the results obtained with the *dnE47* construct (Figure 1D–F) and suggest that E-proteins control the differentiation of NSCs, and influence the cycling behavior of their progeny.

**Figure 5:**
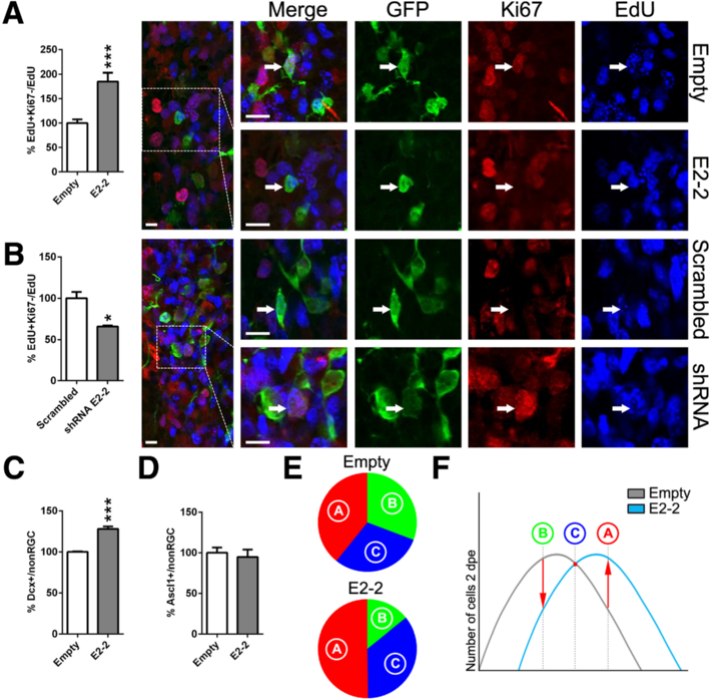
***E2-2 *alteration influences cell cycle exit of progenitors *in vivo*. (A)***E2-2* overexpression increased cell cycle exit (EdU^+^Ki67^–^/EdU^+^) among the progenitor cell population (non-RGC) compared to control conditions at 2 dpe (100 ± 7.5 vs. 184.9 ± 18.0). Confocal micrographs show representative non-RGCs (GFP^+^) having cycled within 24 h prior to sacrifice (EdU^+^) and having re-engaged (Ki67^+^) or exited the cell cycle (Ki67^–^), respectively. **(B)** In contrast, knock-down of *E2-2* decreased cell cycle exit within the progenitor pool as illustrated by the increased Ki67 immunoreactivity among EdU^+^ non-RGCs, when compared to control conditions (Scrambled) (100 ± 7.8 vs. 65.8 ± 1.3). **(C, D)***E2-2* overexpression also increased the number of Dcx^+^ neuroblasts (100 ± 0.5 vs*.* 128.1 ± 2.9) **(C)**, whereas the number of Ascl1^+^ type-C cells remained unchanged (100 ± 6.4 vs. 94.6 ± 9.2) **(D)**. **(E)** Percentage of cell type composition (i.e., type-B = RGC, type-C = Ascl1^+^, type-A = Dcx^+^) upon *E2-2* overexpression, when compared to an empty control plasmid at 2 dpe (30.7 ± 4.6 vs*.* 14.2 ± 2.3, 30.0 ± 1.2 vs*.* 35.6 ± 2.5, 39.2 ± 0.3 vs*.* 50.2 ± 1.8, respectively). **(F)** Summary model: E2-2 orchestrates neurogenesis progression within the murine forebrain. *P* values: *P <0.05; **P <0.01; ***P <0.001. Quantifications were normalized to control conditions **(A–D)**. Scale bars: **A** &**B**, 20 μm.

We next tested whether the changes in proliferation observed following E-protein GoF could be explained by cell cycle exit induction. Dividing cells in S-phase that incorporated 5-ethynyl-2′-deoxyuridine (EdU) 24 h prior to sacrifice, but did not re-enter another round of cell division (i.e., cell cycle exit), would no longer express Ki67. We quantified the number of EdU^+^Ki67^–^/EdU^+^ cells and found a doubling in the number of progenitors that had exited the cell cycle, when *E2-2* was overexpressed (Figure 5A), whereas a smaller number of progenitors exited the cell cycle upon *E2-2* silencing (Figure 5B). Finally, we tested whether *E2-2*-induced cell cycle exit resulted in an abortive differentiation or whether cells appropriately completed their differentiation into Dcx^+^ neuroblasts. We observed a ~30% increase of Dcx^+^ neuroblasts (type-A) within the postnatal forebrain at 2 dpe (Figure 5C, Additional file 1I), which paralleled the reduction of the number of RGCs described above, whereas the number of Ascl1^+^ type-C cells remained unaltered (Figure 5D, Additional file 1J).

Taken together, these results highlight an important role of E-proteins in controlling the timing and progression of NSC differentiation in the postnatal SVZ. Thus, upon *E2-2* overexpression, a concomitant decrease of type-B RGCs and increase of Dcx^+^ type-A cells was observed. These observations, together with the stable number of Ascl1^+^ type-C cells, suggest a more rapid differentiation of *E2-2* overexpressing cells as schematized in Figure 5E,F.

### E47 disruption is not sufficient to mediate the switch of Ascl1-dependent transcription

**Figure 6:**
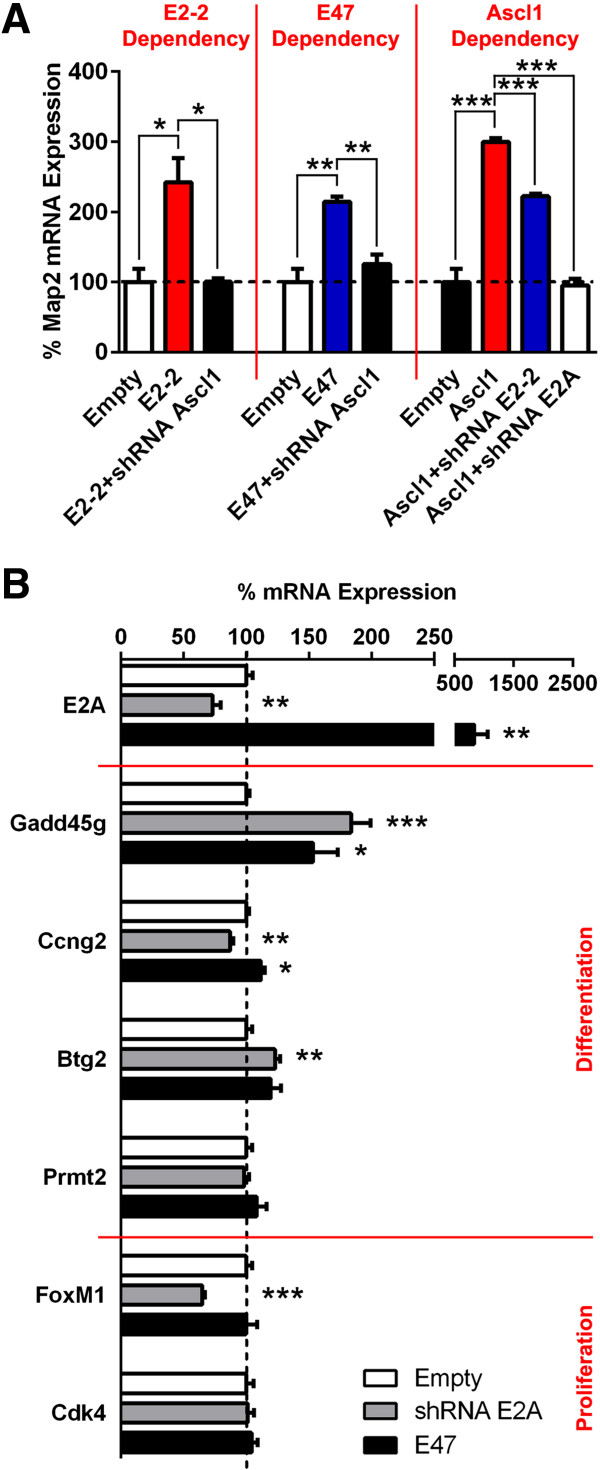
***E47 *fails to trigger Ascl1 to switch from proliferation to differentiation target genes. (A)***Map2* mRNA expression was elevated when *Ascl1* was overexpressed in isolated neurospheres *in vitro* (100 ± 19.1 vs. 299.4 ± 5.8). Concomitant knock-down of *E2-2* or *E2A* using shRNA, respectively, reduced or abolished Ascl1-dependent *Map2* expression (299.4 ± 5.8 vs. 222.2 ± 3.7 or 94.9 ± 9.8, respectively). More strikingly, *Ascl1* silencing decreased *Map2* expression level to control conditions, when *E2-2* (100 ± 19.1 vs. 242.4 ± 34.2, 242.4 ± 34.2 vs. 100.3 ± 4.9) or *E47* (100 ± 19.1 vs. 214.6 ± 7.1, 214.6 ± 7.1 vs. 125.7 ± 13.6) was overexpressed. **(B)** Although significant expression changes could be detected, E-protein gain-of-function did not systematically promote activation of Ascl1 target genes mediating cell cycle arrest. Inversely, E-protein silencing did not activate Ascl1 target genes mediating cell cycle progression. *P* values: *P <0.05; **P <0.01; ***P <0.001. All quantifications were normalized to control conditions.

E-proteins induce NSC differentiation by transactivation of bHLH proneural proteins. Among those is Ascl1, expressed mainly by progenitor cells in the postnatal forebrain [19,38]. We confirmed the interdependence of E-proteins and *Ascl1* by NSC differentiation induction, combining GoF and LoF experiments in isolated progenitors *in vitro. Ascl1* knock-down resulted in a complete loss of *Map2* mRNA transcription, when *E2-2* or *E47* were concomitantly overexpressed. This confirms that E-protein activity is largely dependent on *Ascl1* expression. On the other hand, *Ascl1* is still able to exert its function although to a lesser extent upon silencing of *E2-2*, but not after silencing *E2A,* illustrating both redundancy but also a strong dependency on *E2A* expression in SVZ-derived progenitor cells (Figure 6A).

The bHLH protein Ascl1 has recently been shown to control proliferation and differentiation in neural progenitors by sequentially activating cell cycle progression and cell cycle arrest genes in a context-dependent manner [22]. Therefore, we next assessed whether modifications in E-protein expression levels that occur during neural progenitor cell differentiation could mediate this molecular switch. *E2A*, in the form of *E47*, known to be the main E-protein binding to Ascl1 [21], was overexpressed or silenced in isolated neurosphere cultures and the level of expression of Ascl1 targets, reported to be differentially expressed during cell cycle progression (*FoxM1*, *Cdk4*) and cell cycle arrest (*Gadd45g*, *Ccng2*, *Btg2*, *Prmt2*) [22], was measured by RT-qPCR. No consistent change in expression of genes differentially targeted by Ascl1 was detected following *E2A* GoF or LoF (Figure 6B).

In summary, the divergent context-dependent function of Ascl1 in promoting cell proliferation and in triggering differentiation, as described recently [22], is apparently independent of E-protein activity. Rather, these findings propose that E-protein upregulation during NSC differentiation is likely to act in parallel with other signaling pathways to fine-tune differentiation progression in an Ascl1-dependent manner [39].

## Discussion

In the present study, we demonstrate enriched expression of E-proteins in postnatal germinal regions of the murine forebrain and highlight their key role as transcriptional orchestrators of postnatal SVZ neurogenesis.

bHLH family members are known to regulate a variety of developmental programs in several tissues of the organism, such as myogenesis, hematopoiesis, and pancreatic and heart development, as well as neurogenesis [5]. In the CNS, proneural Class II proteins are the most studied members of the bHLH family. They were shown to be indispensable and sufficient to induce neurogenesis in drosophila [40], *C. elegans*[41,42], and mice [43]. During telencephalic development, *Neurog2* and *Ascl1* confer an opposing dorso-ventral expression gradient to specify distinct neuronal populations [44].

However, less is known regarding Class I bHLH protein expression patterns and their role during neurogenesis. In drosophila, *Daughterless* (E-protein homolog) knock-out results in defective precursor differentiation [45,46]. In contrast, deletion of single Class I bHLH protein members in rodents led to no gross anatomical phenotype [26] or spatially restricted developmental deficits [47]. Deletion of multiple E-proteins resulted in stronger phenotypes highlighting some degree of redundancy in between these genes [26]. Our co-expression experiments confirmed the potential interchangeability of E-proteins, since *E2-2*, *E47*, and *HEB* were equally potent in transactivating Ascl1, even though the LoF analysis revealed Ascl1 to preferentially bind E47. Sequence analysis studies revealed extremely high sequence conservation within, as well as beyond, the HLH domains supporting possible redundancy among individual E-proteins [23]. Indeed, functional replacement of the mouse *E2A* gene with an *E2A* promoter-driven human *HEB* cDNA rescued B-cell commitment and differentiation during B-lymphopoiesis [37].

Furthermore, expression studies have proposed a regulation of E-protein transcription during development, with a gradual restriction to germinal regions in the CNS [25], supporting key neurogenesis functions. Our ISH combined with RT-qPCR gene expression analysis confirmed an enrichment of all E-protein transcripts in postnatal and adult germinal regions of the forebrain when compared to non-neurogenic regions. Notably, these expression experiments uncovered *E2-2* as the most abundant E-protein in the SVZ, followed by a less prominent expression for *E2A* and *HEB*. Additionally, E-proteins seem not only to be restricted spatially but also temporally during lineage progression. More differentiated progenitors exhibited increased transcription levels *in vitro* as well as *in vivo*, further implicating a specialized function in maintaining proper neurogenesis behavior. Altogether, these results highlight a complex regulation of E-protein expression in germinal regions of the postnatal forebrain.

The dramatic blockade of NSC differentiation and subsequent neurogenesis observed following *dnE47* overexpression *in vitro* as well as *in vivo* underlines the importance of a functional bHLH network for lineage progression. Based on these findings, we further aimed at manipulating E-protein expression more concisely in the lateral SVZ using overexpression and shRNA plasmids within an established postnatal electroporation approach [31,32]. Major alterations in progenitor proliferation and cell cycle exit were evident. These results are in agreement with a previously described role of E2A in cell cycle regulation in transiently transfected NIH3T3 fibroblasts, by activating cyclin-dependent kinase inhibitors [27,48,49]. Moreover, Id proteins, known E-protein antagonists (see above), enhance cell cycle duration and prevent cell cycle exit, which was further confirmed with a decreased *cyclinD1* and increased *p27*^*Kip1*^ (cyclin-dependent kinase inhibitor 1B) expression within the VZ of triple knock-out mice [18]. In line with Id protein mediated anchorage to the neurogenic niche [18], E-proteins were also reported to actively repress expression of E-cadherin, a cell adhesion molecule [50,51]. Recent studies in drosophila, revealing a direct transcriptional regulation of *Emc* (Id protein homolog) by *Daughterless*, coupled with the physical sequestration of E-proteins by Class V bHLH members [6], results in an autoregulatory feedback [24,52]. In addition, E-protein expression and therefore lineage progression seems to be initially mediated by extracellular signaling pathways, which is consistent with earlier reports, suggesting a general role for Shh and BMPs in SVZ neurogenesis [53-55]. In drosophila, it was demonstrated that Hh (Shh homolog) and Dpp (BMP homolog) specifically repress Id expression and thus increase E-protein activity [24], ultimately leading to germinal niche detachment and altered cell cycle behavior.

Our findings suggest that the effects of E-proteins on SVZ NSC differentiation are entirely dependent on *Ascl1*, which is expressed in all postnatal SVZ progenitors [19,56]. Knock-down of *Ascl1* entirely abrogated the proneural activity of *E2-2* and *E2A*, demonstrating that E-proteins converge with the same Class II bHLH protein. The incomplete loss of Ascl1 activity when *E2-2* is silenced is likely due to a persistent endogenous E47 activity, as Ascl1 seems to rely more on *E2A* than on *E2-2*, demonstrated by the absence of Ascl1 activity upon *E2A* silencing. Recent findings propose that sustained *Ascl1* expression favors cell differentiation, whereas oscillatory expression correlates more with proliferative cell fates [57]. Given that E-proteins, in competition with Id proteins, tightly regulate Ascl1 protein stability [58] and that Ascl1 has lately been demonstrated to target two subsets of genes, namely proliferation- and differentiation-associated genes [22], we investigated a role for E-proteins in mediating this switch in binding bias. However, in the present study, *E2A* alterations did not induce directed and systematic gene expression changes, at least for the Ascl1 target genes tested, most likely mitigating the probability of E-proteins to induce differentiation through promoting Ascl1 binding and activation of cell cycle arrest genes. This implies that E-proteins are likely to operate in parallel with other signaling pathways to fine tune differentiation progression in an Ascl1-dependent manner [39].

Although our results did not reveal significant differences in the expression pattern of distinct E-proteins during postnatal SVZ neurogenesis, and suggest an interchangeability of E-proteins in the context of Ascl1-induced SVZ NSC differentiation, it should be noted that the dimerization properties of bHLH proteins can be affected at the post-translational level. For example, GSK3β phosphorylation of Neurog2 was described to influence its dimerization with E47 in the developing cortex, thereby affecting its transcriptional activity [59]. Similar mechanisms might be at play in the postnatal SVZ to fine tune the choice of proneural/E-protein-binding partners and regulate individual aspects of NSC differentiation, such as, for example, specification. Previous studies have indeed demonstrated a specific role of E2-2 for the generation of a unique subset of neural progenitors during pontine development [47], despite the presence of other E-proteins. Also, B- and T-lymphocyte specification was shown to be orchestrated by E2A and HEB [60,61]. Whether Class I/II bHLH protein dimerization is dynamically regulated in the postnatal SVZ to influence fate specification is currently unknown and will be the subject of future investigations. Performing such experiments in the postnatal SVZ is particularly attracting due to the regional expression of some proneural proteins as, for example, Neurog2, which shows dorsal enrichment in the postnatal SVZ in a population that gives rise to glutamatergic neurons [62,63].

## Conclusions

In the present work, we demonstrate a diverse expression pattern of Class I bHLH proteins and underpin their underestimated significance in orchestrating early neuronal lineage progression. However, the precise mechanism behind E-proteins regulating NSC differentiation remains to be elucidated, but is unlikely to rely solely on Ascl1 heterodimerization.

## Methods

### Cell culture and *in vitro* nucleofection

The mouse embryo-derived and immortalized RGC line NS5 was cultured as described earlier [28] and was nucleofected following the manufacturer’s instructions using a 4D-nucleofector device with SG solution (Lonza, Basel, Switzerland) and the EN150 program with a maximum of 0.5 μg of plasmid DNA per 1 × 10^6^ cells. NS5 cell differentiation was achieved by plating cells into polyornithin-coated (0.001% in H_2_O, Sigma-Aldrich, St. Louis, MO, USA) and laminin-coated (20 μg/mL in H_2_O, Life Technologies, Carlsbad, CA, USA) flasks in D1 medium (Euromed-N stem cell medium; Lucerna Chem, Lucerne, Switzerland) supplemented with 1% penicillin/streptomycin, 0.25% L-glutamine, 1% B27 (Life Technologies), 0.5% N2 (Life Technologies), and 5 ng/mL FGF2 (Peprotech, Hamburg, Germany) [64].

Neurosphere-forming progenitor cells were isolated from the SVZ of P4 aged animals. The SVZ was microdissected and the tissue digested with a solution of DNAse I (Worthington, Lakewood, NJ, USA), Papain (Worthington), and Dispase II (Roche, Basel, Switzerland), subsequently triturated and filtered through a 70 μm cell strainer (BD Biosciences, Franklin Lakes, NJ, USA). Cells were plated into DMEM/F12 medium supplemented with 1% N2, EGF, FGF2 (both 20 ng/mL, Peprotech), heparin (1 μg/mL), and 1% penicillin/streptomycin. For nucleofections, a mouse neural stem cell solution (Lonza), program DS113, and a maximum of 1 μg of plasmid DNA per 1 × 10^6^ cells were used.

Both NS5 and progenitor cells were subjected to RNA isolation and subsequent RT-qPCR 24 to 48 h post-nucleofection.

### Animals and postnatal electroporation

All animal experiments were performed in agreement with the Canton of Zurich veterinary office guidelines (authorization 182/2011). All mice used in this study were of the CD1 (Swiss mice) strain and were obtained from the Charles River laboratory.

Electroporation of RGCs lining the lateral ventricle was performed on postnatal-day-2 mice (P2), as described previously [31,32]. In brief, following anesthesia by hypothermia, P2 pups were injected with 2 μL of plasmid solution (5 μg/μL in PBS). Fast Green 1% (Sigma-Aldrich) was used to assess accuracy of the intra-ventricular injections. Successfully injected mice were subjected to five electrical pulses (95 V, 50 ms, separated by 950 ms intervals) using the ECM 830 BTX electroporator (Harvard Apparatus, Holliston, MA, USA) and tweezer electrodes (5 mm diameter; BTX Tweezertrodes, Harvard Apparatus) coated with conductive gel (Signa gel, Parker Laboratories, Fairfield, NJ, USA). For proliferation studies, 50 mg/kg 5-ethynyl-2′-deoxyuridine (EdU) were administered subcutaneously 24 h prior to sacrifice.

### Tissue processing

Two days post-electroporation, mice were deeply anesthetized (150 mg/kg pentobarbital, i.p.) and transcardially perfused with Ringer solution supplemented with heparin (250 mg/L, Sigma-Aldrich) followed by 4% paraformaldehyde (PFA) (Sigma-Aldrich). Then, 50 μm thick vibratome sections (Leica, Wetzlar, Germany) were collected as a series of six encompassing the entire rostro-caudal extent of the lateral ventricle. Sections were stored at –20°C in antifreeze solution (25% Glycerol, 25% Ethylenglycol and 50% 0.1 M PB).

### Immunostaining

All immunostainings were performed on a complete series of free-floating sections. Blocking and permeabilization were achieved by incubating the sections for 2 h in 0.1 M PB supplemented with 0.4% Triton-X100 and a TNB buffer (thereafter mentioned as 0.4% TNB-TX100) consisting of 0.05% Casein (Sigma-Aldrich), 0.25% bovine serum albumin (Sigma-Aldrich), and 0.25% Topblock (Lubio Science, Lucerne, Switzerland) at room temperature. Afterwards, primary antibody incubations were performed in 0.4% TNB-TX100 at 4°C overnight (M α Ki67, 1:300 (BD Biosciences); Rb α Ki67, 1:500 (MM France, Francheville, France); G α Dcx, 1:300 (Santa Cruz Biotechnology, Santa Cruz, CA, USA); Ch α Vimentin, 1:1000 (Millipore, Darmstadt, Germany); and M α Ascl1, 1:300 (BD Biosciences)). Then, incubation with species-matched secondary antibodies (Life Technologies) was performed for 2 h at 4°C in 0.4% TNB-TX100. GFP signal was amplified using a biotin-conjugated secondary antibody against the GFP primary antibody (Ch α GFP, 1:1,000 (Aves Labs, Tigard, OR, USA)). In addition, the tissues were incubated for 15 min with a biotin-specific streptavidin Alexa 488 complex. Finally, sections were counterstained using 4′,6-diamidino-2-phenylindole (DAPI, Sigma-Aldrich) and coverslipped with antifading mounting medium (Vectashield, Vector Labs, Burlingame, CA, USA).

For nuclear stainings, an antigen retrieval protocol was applied. Sections were incubated with 10 mM tri-sodium citrate buffer (+0.05% Tween 20, pH 6.0) for 30 min at 80°C and 20 min at room temperature.

Detection of EdU incorporation was performed using the Click-it EdU system (Life Technologies) according to the manufacturer’s instructions.

### *In situ* hybridization

*In situ* RNA hybridization was performed as previously described [65]. After dissection, postnatal or adult brains were fixed in 4% PFA at 4°C for 48 or 24 h, respectively, cryoprotected in 30% sucrose at 4°C overnight, cryosectioned at 20 μm on consecutive slides, and stored at −80°C. Sections were acetylated with 0.1 M triethanolamine and 2.5 μL/mL acetanhydride for 10 min and additionally fixed in 4% PFA at RT for 15 min before prehybridization for 4 h followed by 1 to 2 μg/mL probe hybridization for 16 h at 65°C. The antisense *E2-2* [NM_013685], *E2A* [NM_011548], and *HEB* [NM_011544] probes were derived from plasmid constructs, whereas linearized templates were transcribed using a T7 RNA polymerase (Thermo Fisher Scientific, Waltham, MA). Anti-DIG FAB (1:2000 (Roche)) immunostaining was applied at 4°C overnight and subsequently developed in buffer containing BCIP (175 μg/mL) and NBT (100 μg/mL) for 12-48 h.

### Microdissection and MAC sorting

To determine the spatio-temporal E-protein expression pattern, the SVZ or the striatum were microdissected from mice aged P6 as described in previously published protocols [66]. In brief, NSCs and neuroblasts were isolated by magnetic affinity cell sorting (MACS) using conjugated antibodies against the transmembrane proteins Prominin-1, also known as CD133, and PSA-NCAM. Dissected tissues were pooled in Hanks buffered salt solution (Life Technologies) and dissociated using a trypsin-based kit following manufacturer’s guidelines (Miltenyi Biotec, Bergisch Gladbach, Germany). Then, anti-Prominin-1 or anti-PSA-NCAM antibodies conjugated with magnetic beads (Miltenyi Biotec) were applied to the cell suspension for 30 min at 4°C before they were passed through MACS separator columns (Miltenyi Biotec). Sorted cells were snap-frozen for subsequent RT-qPCR. One litter represents 1 ‘n’ number.

### Gene expression analysis

RNA was extracted, processed, and amplified from tissues/cells as previously described [32]. For RT-qPCR, 20 ng of cDNA were loaded with 5 μm of forward and reverse primers (*Map2*: fw_GCTGAAGCTGTAGCAGTCCTGAA, rv_GTGTTGGGCTTCCTTCTCTTGT; *E2-2*: fw_GGGAAAGCCCTAGCTTCGAT, rv_CCCACAGGACTTGAAGGATTG; *E2A*: fw_TGGGCTCTGACAAGGAACTGA, rv_CCGGCTCTTCCCATTGG; *HEB*: fw_CCATCCCCAAATTCTGACGAT, rv_GCTGGCTCATCCCATTCG; *Dcx*: fw_CTGACTCAGGTAACGACCAAGAC, rv_TTCGAGGGCTTGTGGGTGTAGA; *CD24*: fw_ACATCTGTTGCACCGTTTCCCG, rv_CAGGAGACCAGCTGTGGACTG; *GFAP*: fw_GCAGAAGCTCCAAGATGAAAC, rv_CCTTTCTCTCCAAATCCACAC; *Id1*: fw_CCTAGCTGTTCGCTGAAGGC, rv_CTCCGACAGACCAAGTACCAC; *ABCG2*: fw_CTCAACCTGCCCATTTCAAATGCT, rv_GTTGGAAGTCGAAGAGCTGCTGAGA; *Gadd45g*: fw_CTGCTGTGAGAACGACATTG, rv_GGTCCTTCCATGTGTCCTCA; *Ccng2*: fw_CTACAGTGTTCCTGAGCTGC, rv_GTCTGAGCCACTTGGAAGTC; *Btg2*: fw_GCGAGCAGAGACTCAAGGTT, rv_CGGATACAGCGATAGCCAGA; *Prmt2*: fw_CCACAGCAAGGTGCTCTTCT, rv_TGCATGGCTCAGAGAGACAG; *FoxM1*: fw_CTACACTTGGATTGAGGACC, rv_CCATTGGCAGATGTCTCTCG; *Cdk4*: fw_GGACCTGAAGCCAGAGAACA, rv_AGATACAGCCAACGCTCCAC), SYBR green mastermix (Roche), and DNAse/RNAse free H_2_0 (Sigma-Aldrich) onto 96-well plates for LightCycler 480 (Roche). All samples were run in duplicates or triplicates, whereas *glyceraldehyde-3-phosphate dehydrogenase* (*GAPDH*: fw_CGACTTCAACAGCAACTCCCACTCTTCC, rv_TGGGTGGTCCAGGGTTTCTTACTCCTT) was used as reference gene. Relative gene expression was determined using the ^ΔΔ-^CT method [67].

### Analysis

Images were acquired with a laser scanning confocal microscope (Olympus IX 81, Olympus, Tokyo, Japan) equipped with a 20× or 40× objective, or with an epifluorescence microscope (Leica DM5500 B, Leica). Quantifications following *in vivo* electroporations are based on an average cell count of 254 successfully transfected cells per animal (n ≥5). Each animal represented 1 ‘n’ number. Morphological criteria to define RGCs were bipolar morphology and apical connection to the LV lumen. Morphological criteria to define non-RGCs were absence of apical and basal processes (i.e., roundish appearance), and absence of contact to the LV lumen [31,32].

All data are expressed as mean and standard error of the mean (±SEM). *P* values were determined using unpaired *t*-test or two-way ANOVA followed by Dunnett’s *post hoc* test (GraphPad Prism v5 software, San Diego, CA, USA).

## Abbreviations

bHLH: Basic-helix-loop-helix; CNS: Central nervous system; *dnE47*: Dominant-negative E47; dpe: Days post-electroporation; EdU: 5-ethynyl-2′-deoxyuridine; GFP: Green fluorescence protein; GoF: Gain-of-function; ISH: *In situ* hybridization; LoF: Loss-of-function; LV: Lateral ventricle; MACS: Magnetic affinity cell sorting; non-RGC: Non-radial glial cell; NSC: Neural stem cells; PFA: Paraformaldehyde; RGC: Radial glial cell; RMS: Rostral migratory stream; RT-qPCR: Real-time quantitative polymerase chain reaction; SVZ: Subventricular zone.

## Competing interests

The authors declare that they have no competing interests.

## Authors’ contributions

BF, KA, SR, and MF carried out the experimental work. AHC contributed expertise for the subcloning of plasmids. BF and OR wrote the manuscript. OR conceived the study. All authors read and approved the final manuscript.

## Supplementary Material

Additional file 1 **(A)** In contrast to the blockade of *Ascl1-*induced neuronal differentiation shown in Figure 1C, *dnE47* overexpression alone did not affect Map2 expression i*n* NS5 cells kept in proliferative culture conditions (100 ± 1.0 vs*.* 93.9 ± 5.3). **(B)** Ki67 immunoreactivity revealed an increased non-RGC proliferation after *dnE47,* when compared to empty control conditions, as shown in Figure 1E. **(C & D)** Antigenic characterization of Ki67^+^ non-RGCs, demonstrated that both Ascl1^+^ type-C (C, Figure 2C) and, to a lesser extent, Dcx^+^ type-A cells (D, Figure 2C) proliferate. **(E–G)** Representative immunostainings for detected changes in the expression of cell type specific markers; i.e., RGC = Vimentin^+^**(E)**, type-C = Ascl1^+^**(F)**, type-A = Dcx^+^**(G)**, following *dnE47* expression, as quantified in Figure 2D–G. **(H)** Absence of cross-reactivity between shRNAs against *E2A* and *E2-2* transcripts (see also Figure 4B). *E2A* mRNA expression was not reduced when shRNA against *E2-2* was applied *in vitro* (100 ± 7.6 vs*.* 111.5 ± 1.1). Similarly, *E2-2* mRNA expression was not reduced when shRNA against *E2A* was used (100 ± 2.7 vs*.* 148.6 ± 8.7). **(I, J)**, Representative immunostainings for Dcx and Ascl1 illustrating an increased Dcx expression (**I**, quantified in Figure 5C) and unaltered *Ascl1* expression (**J**, quantified in Figure 5D), when *E2-2* was overexpressed. Arrows identify representative cells for each experimental condition. Quantifications were normalized to control conditions. Scale bars: B–G, I, J, 20 μm. Click here for file
