# Supplementary figures and images for: E-proteins orchestrate the progression of neural stem cell differentiation in the postnatal forebrain
Source: Neural Dev. 2014 Oct 29;9:23. doi: 10.1186/1749-8104-9-23 (PMC4274746; doi:10.1186/1749-8104-9-23)

**A**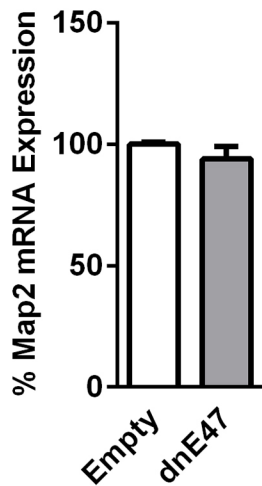**B**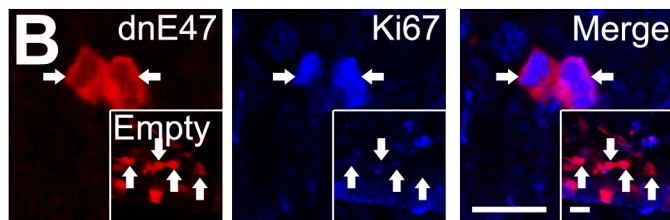**C**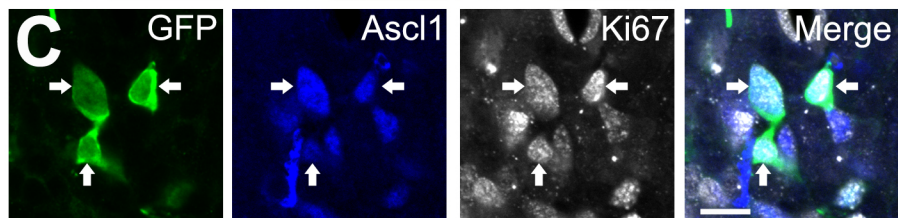**D**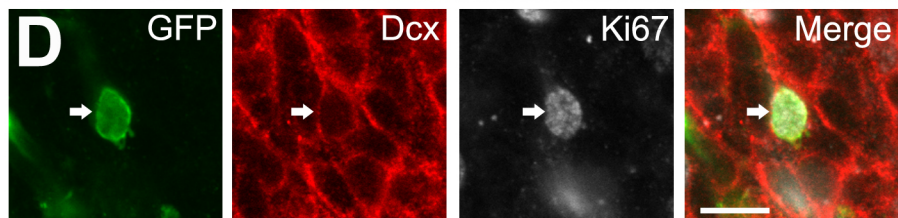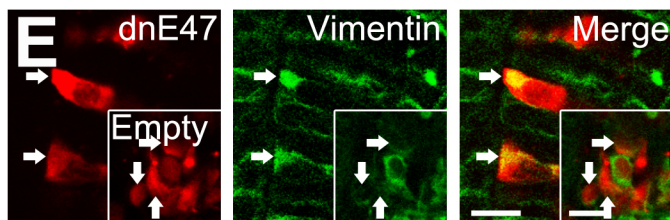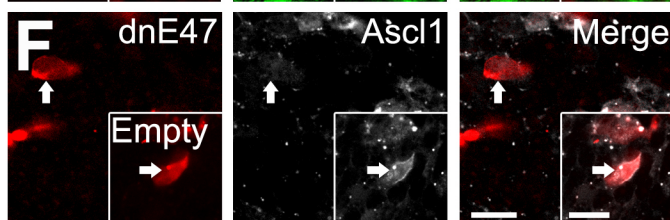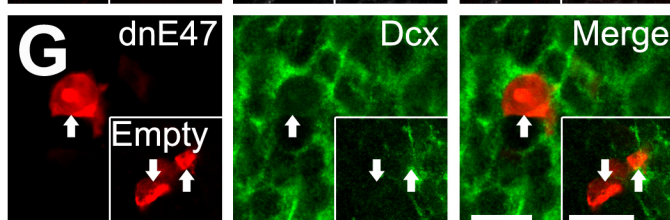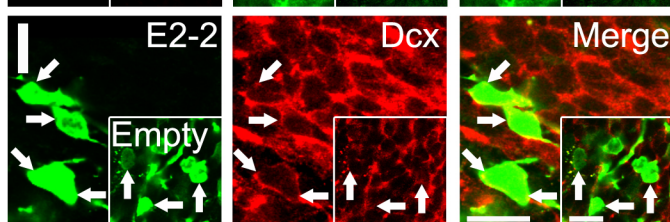**H**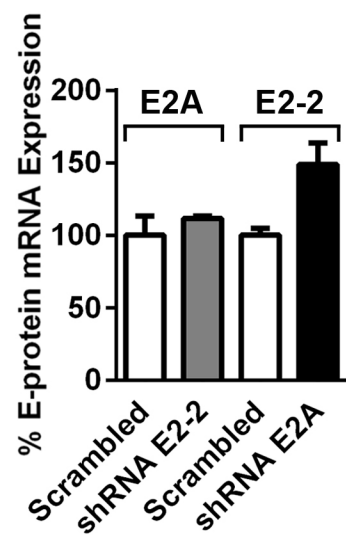**J**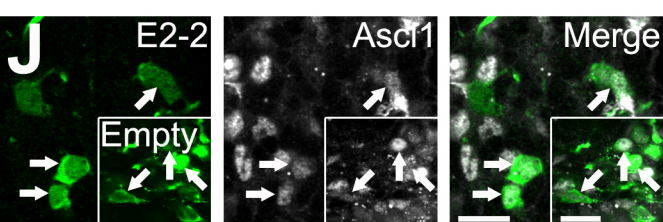

Supplement: Additional file 1 — (A) In contrast to the blockade of Ascl1-induced neuronal differentiation shown in Figure 1C, dnE47 overexpression alone did not affect Map2 expression in NS5 cells kept in proliferative culture conditions (100 ± 1.0 vs. 93.9 ± 5.3). (B) Ki67 immunoreactivity revealed an increased non-RGC proliferation after dnE47, when compared to empty control conditions, as shown in Figure 1E. (C & D) Antigenic characterization of Ki67+ non-RGCs, demonstrated that both Ascl1+ type-C (C, Figure 2C) and, to a lesser extent, Dcx+ type-A cells (D, Figure 2C) proliferate. (E–G) Representative immunostainings for detected changes in the expression of cell type specific markers; i.e., RGC = Vimentin+(E), type-C = Ascl1+(F), type-A = Dcx+(G), following dnE47 expression, as quantified in Figure 2D–G. (H) Absence of cross-reactivity between shRNAs against E2A and E2-2 transcripts (see also Figure 4B). E2A mRNA expression was not reduced when shRNA against E2-2 was applied in vitro (100 ± 7.6 vs. 111.5 ± 1.1). Similarly, E2-2 mRNA expression was not reduced when shRNA against E2A was used (100 ± 2.7 vs. 148.6 ± 8.7). (I, J), Representative immunostainings for Dcx and Ascl1 illustrating an increased Dcx expression (I, quantified in Figure 5C) and unaltered Ascl1 expression (J, quantified in Figure 5D), when E2-2 was overexpressed. Arrows identify representative cells for each experimental condition. Quantifications were normalized to control conditions. Scale bars: B–G, I, J, 20 μm. [file 1749-8104-9-23-S1.pdf]
